# A lightweight Color-changing melon ripeness detection algorithm based on model pruning and knowledge distillation: leveraging dilated residual and multi-screening path aggregation

**DOI:** 10.3389/fpls.2024.1406593

**Published:** 2024-07-22

**Authors:** Guojun Chen, Yongjie Hou, Haozhen Chen, Lei Cao, Jianqiang Yuan

**Affiliations:** ^1^ Qingdao Institute of Software, College of Computer Science and Technology, China University of Petroleum (East China), Qingdao, China; ^2^ Faculty of Light Industry, Qilu University of Technology, Jinan, China; ^3^ State Key Laboratory of Biobased Material and Green Papermaking, Shandong Academy of Sciences, Jinan, China

**Keywords:** Color-changing melon, multi-scale feature fusion, model pruning, knowledge distillation, YOLOv8s

## Abstract

Color-changing melons are a kind of cucurbit plant that combines ornamental and food. With the aim of increasing the efficiency of harvesting Color-changing melon fruits while reducing the deployment cost of detection models on agricultural equipment, this study presents an improved YOLOv8s network approach that uses model pruning and knowledge distillation techniques. The method first merges Dilated Wise Residual (DWR) and Dilated Reparam Block (DRB) to reconstruct the C2f module in the Backbone for better feature fusion. Next, we designed a multilevel scale fusion feature pyramid network (HS-PAN) to enrich semantic information and strengthen localization information to enhance the detection of Color-changing melon fruits with different maturity levels. Finally, we used Layer-Adaptive Sparsity Pruning and Block-Correlation Knowledge Distillation to simplify the model and recover its accuracy. In the Color-changing melon images dataset, the mAP0.5 of the improved model reaches 96.1%, the detection speed is 9.1% faster than YOLOv8s, the number of Params is reduced from 6.47M to 1.14M, the number of computed FLOPs is reduced from 22.8GFLOPs to 7.5GFLOPs. The model’s size has also decreased from 12.64MB to 2.47MB, and the performance of the improved YOLOv8 is significantly more outstanding than other lightweight networks. The experimental results verify the effectiveness of the proposed method in complex scenarios, which provides a reference basis and technical support for the subsequent automatic picking of Color-changing melons.

## 1 Introduction

Color-changing melon, a kind of fruit that turns from green to red on the surface of its skin when it matures, belongs to the Cucurbitaceae family of vines, and the fruit is thick in the middle and thin at both ends, resembling a mouse, so it is also known as the mouse melon. It is suitable for planting in the garden, both ornamental and edible. When cultivated on the plantation, workers will plant the seedlings in the hanging soil, and when the plant grows up, the vine climbs all over the shelves. The fruit naturally hangs down with a reddish color for a good ornamental appearance. At the same time, Color-changing melons have a high yield, and a single plant can get about 200 fruits in its lifetime. Therefore, after some ornamental fruits are left behind, most of the remaining immature fruits are picked and used in stir-fries or soups for a refreshing flavor. A Color-changing melon plant can produce fruit for up to five consecutive months. Due to the varying maturity periods of the fruits, in the current production environment, the immature fruits are mainly picked by hand. Fruits picked too early have a rugged quality and poor flavor, while fruits picked too late lose their food value and affect profitability (Camposeo et al., 2013). If you rely only on workers, you need to pick several times, which is too time-consuming and inefficient (Yang et al., 2023). Meanwhile, the fruits are all growing on 3-meter-high shelves, and picking operations that do not meet safety norms increase the risk of worker injury. In response to these problems, we believe robotic arms (Kang et al., 2020) can be developed to automatically pick fruits that meet standards. This can alleviate the problem of labor shortage in agricultural production (Clark et al., 2018) and, at the same time, ensure the quality of picking, improve productivity, and ensure the safety of workers. However, there are still some difficulties in robotic picking technology, and the critical step is the localization and judgment of the fruit. The study of how to realize accurate target detection is a prerequisite for automatic picking work.

In the early field of target detection, researchers designed detection algorithms based on the fruit’s color, shape, and texture. However, for fruits whose fruit color is similar to that of leaves, such as cucumbers, it is impossible to distinguish the fruit from the background by relying on shape alone. Therefore, researchers usually use morphology in conjunction with other methods in the process of designing algorithms. For example, Dorj et al (Dorj et al., 2017). designed algorithms to detect citrus based on color and shape features. However, traditional algorithms are only designed for a specific scene. If the interference of environmental factors such as light changes is considered (Zhang et al., 2022), the detection effect on the target will be significantly reduced. Traditional machine learning algorithms have some improvements in detecting fruits. However, they still have similar problems: they often need to limit the types of features to compress the feature space (Chaudhari and Waghmare, 2022), they cannot learn high-dimensional features directly, and they are not robust and generalized enough to face a variety of complex scenes.

As science continues to develop, Convolutional Neural Networks (CNNs) have overcome the limitations of traditional machine learning and demonstrated excellent performance (Krizhevsky et al., 2017). CNN-based machine vision has been increasingly widely used in agriculture (Kamilaris and Prenafeta-Boldú, 2018), and the resulting deep-learning networks are continuously penetrating the field of Computer Vision. With the structure of CNNs as the Backbone, the model extracts rich feature information and dramatically improves the accuracy of detection, while the high-dimensional features processed by multi-layer convolution further enhance the generalization of different application scenarios. Classical target detection algorithms consist of a classification process and a localization process, and these algorithms can be classified into two-stage detection algorithms and one-stage detection algorithms based on whether they produce candidate regions. The R-CNN family of networks are representative algorithms for two-stage detection, which first generate candidate regions and then perform the target classification task and the target localization task separately, for instance, Faster R-CNN (Ren et al., 2015) and Mask R-CNN (He et al., 2017). Mu et al (Mu et al., 2020). used Faster R-CNN incorporating transfer learning and achieved a mAP of 87.83% on a homemade immature tomato dataset. Jin et al (Suddapalli and Shyam, 2021). used Mask R-CNN to segment diseased portions of vegetables and fruits, which was used in place of the manual screening process.

In contrast, one-stage detection algorithms have faster detection speeds and such algorithms are represented by SSD (Liu et al., 2016) and the YOLO family (Redmon et al., 2016). The YOLO family has iterated many versions through continuous development (Terven et al., 2023), with progressively improved extensibility and generalization, and is now widely used in detecting fruits and diseases in agriculture (Shi et al., 2020; Suo et al., 2021; Nan et al., 2023; Zhu et al., 2024). Liang et al (Liang et al., 2020). combined YOLOv3 and UNet to detect lychee under nighttime conditions. YOLOv3 suffers from the problem of a relatively complex model structure. Therefore, subsequent research has also focused on lightweight target detection algorithms. Li et al (Li et al., 2021). modified the YOLOv4-Tiny model to design a detection algorithm for corn kernel breakage during harvesting and provide parameters for the combined harvester while working. Zeng et al (Zeng et al., 2023). used the MobileNetv3 network to replace Backbone in YOLOv5 while optimizing the training hyperparameters. They constructed a lightweight model successfully deployed to cell phones to detect tomatoes’ maturity. Nouaze et al (Nouaze and Sikati, 2023). introduced the FEature architecture in YOLOv7, which was used to combine various pieces of information in the feature space and increase the model’s recognition accuracy for both healthy and diseased apples, and its recognition accuracy with a mAP of 89.30%.

Although the above-improved algorithms have made progress in model lightweight, they only pursue the simplification of network structure when they face complex real-world scenarios, such as backlighting, overlapping fruits, dense fruits, and fruits being occluded by other objects, many of the target detection algorithms that have been lightweight are limited by the small number of parameters and computation, their robustness is not ideal, and they often miss and misdetect, which makes it difficult to cope with the detection in complex scenes. Therefore, in response to the challenge, most lightweight models are less robust when facing complex scenes. In contrast, high-accuracy models suffer from a more complex network structure; this paper constructs an improved model based on YOLOv8s as well as a series of subsequent processing of the model, which can be used for the real-time detection task of picking robots and low-cost edge devices in complex natural environments under the premise of guaranteeing the detection accuracy.

These combined algorithms can effectively improve the shortages of sizeable computational cost and excessive memory occupation during the model deployment while maintaining a high accuracy rate, providing technical support for the subsequent automatic harvesting.

The main contributions and innovations of this study are summarized as follows:

(1) We created a dataset of Color-changing melon figures using manual annotation, and the fruits in various real scenarios were considered in the shooting process.
(2) In order to improve the accuracy of target detection in complex scenes while maintaining the lightweight structure of the model, we first designed the DWR-DRB module to replace the Bottleneck in the C2f module to increase the receptive field without increasing the depth of the network, to enrich the multi-scale contextual information extracted by Backbone. Then, we constructed the HS-PAN architecture, which adopts multi-level feature fusion to aggregate multi-scale features and can effectively focus on the fruits that are interfered with by background factors.
(3) After the model was trained, we used layer adaptive sparsity pruning on the model, and the pruned model computed only one-third of the original FLOPs. Then, using the improved model trained in advance as the teacher model, the pruned model is distilled using block correlation knowledge distillation, and the student model does not increase the network complexity. At the same time, the recognition performance is further improved, which helps the model to be deployed on mobile terminals or embedded devices with limited resources.
(4) We conducted a series of comparison experiments related to Color-changing melon dataset detection. We first conducted a comparison of the model recognition effect before and after improvement, then designed an ablation experiment, next compared the number of each channel before and after model pruning and the effect of different scales of teacher models on the distillation effect, and finally compared our improved algorithm with other lightweight algorithms to demonstrate the difference in performance between different algorithms.

The rest of the paper is organized as follows. Part II discusses the processing flow of the Color-changing melon dataset and the improved YOLOv8s model and also describes the model pruning and knowledge distillation methods used. Part III explains the experimental setup and evaluation metrics and discusses the results of the various types of comparisons. Part IV summarizes.

## 2 Materials and methods

### 2.1 Data acquisition

**Figure 1:**
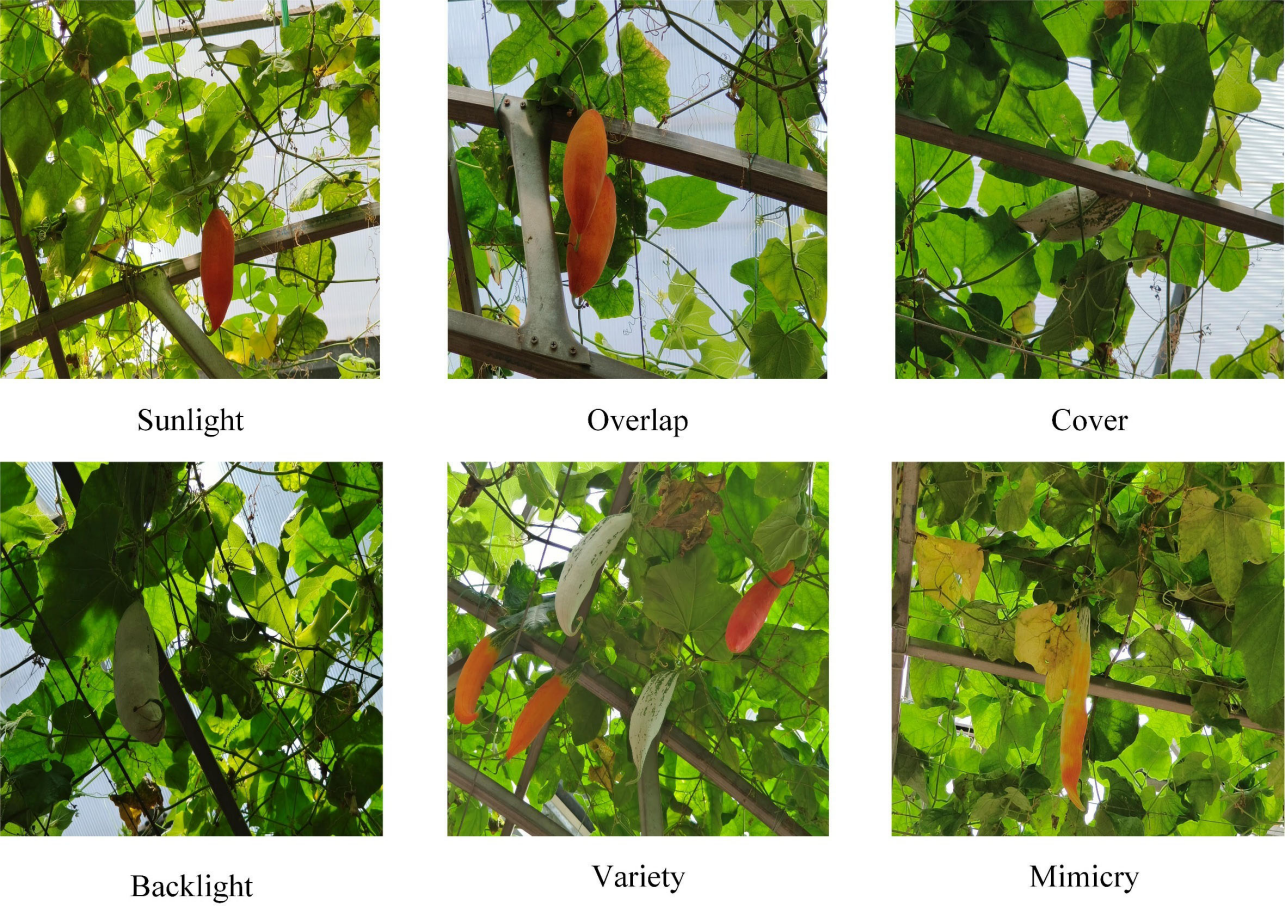
Images captured under different scenes.

The Color-changing melons dataset utilized in our research was collected from a vegetable science and technology park in Shouguang City, Shandong Province (36°51′N, 118.49′E), and photographed during July 2023, every day from 10:00 a.m. to 4:00 p.m. All images were obtained using the Sony IMX 866 rear camera of the Vivo X80 smartphone under natural lighting conditions. The shooting distance ranged from 0.8 meters to 1.2 meters. The images consider variations in factors such as shooting angle, lighting, and fruit overlap. After filtering out low-quality images, such as overexposure and severe blurring, 1240 images were finally obtained and archived in a JPG file type of 4032×3024 pixels. **Figure 1** displays the sample data obtained from various shooting scenarios.

### 2.2 Data labeling

In the task of detecting the maturity of Color-changing melons, the maturity of Color-changing melons was classified into green immature, orange semi-mature, and red mature stages based on the color of the fruit surface in accordance with agricultural harvesting requirements. Some of the green immature stages have fine stripes present on the surface of the fruit. When the surface of the fruit fades from green to orange starting from the top, this enters the semi-mature stage. The immature stage is reached when the color of the fruit surface gradually deepens until it turns completely red. During actual harvesting, a small number of semi-mature and mature fruits are used for ornamental purposes as well as seed reserves, while most of the immature fruits are picked for consumption. The use of algorithms to obtain information on the maturity of the fruit helps to provide a basis for judgment of the picking work of the robot. Without affecting the recognition accuracy, we set the images in the Color-changing melons dataset uniformly at 640×640 pixels and randomly divided them into the training set, validation set, and test set according to the ratio of 8:1:1 . The images of the three maturity levels of fruits are uniformly distributed in each set without intersecting each other. There are 992 images in the training set and 124 in the validation and test sets, respectively. Then, all the images are labeled using LabelImg, and the labels are saved in txt format and converted to xml format for easy training and testing.

### 2.3 Data augmentation

**Figure 2:**
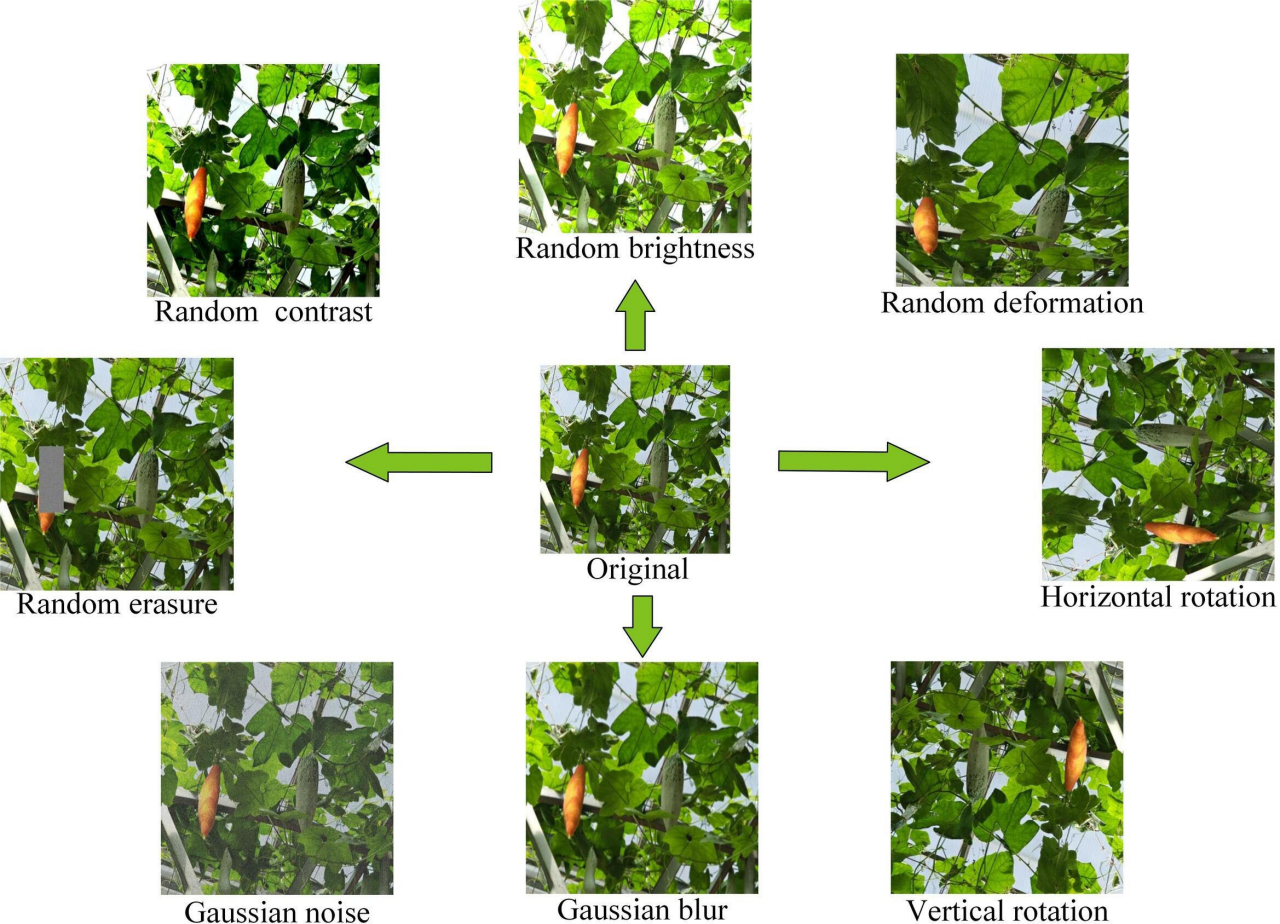
Examples of various data augmentation techniques.

To improve the trained network’s effectiveness and enhance the model’s robustness, data enhancement methods are used to increase the number of images in the training part to avoid overfitting. With the help of the Augmentor tool, we performed operations such as flipping, brightness adjustment, warping distortion, and adding noise to the images, 150 images were obtained for each enhancement method, and finally, the training set was expanded to 2142 images. **Figure 2** provides an example of each data augmentation technique. It is worth noting that in cases where it is difficult to obtain a large number of labeled fruit figures, in addition to the offline data augmentation approach used in the paper, it is good to consider using FSL (Few-Shot Learning) or Meta-learning to help the model improve its generalization ability. These two approaches provide practical tools for dealing with data scarcity in resource-constrained environments. Meta-learning learns from a few crucial fruit samples and thus adapts quickly to different fruit maturity stages. Further, it enhances adaptation to new tasks by learning multiple related tasks and constructing similar sets between different tasks. Few shot learning is a learning strategy to improve the model’s ability to generalize to new tasks with fewer supervised samples, and it usually utilizes prior knowledge to simplify the sample features. In practical agricultural applications, these two methods can be combined to train the base model through meta-learning first and then use few shot learning to fine-tune the model and improve its generalization in the case of limited samples to apply the target detection technology more widely to agricultural automated picking systems.

### 2.4 YOLOv8

Until 2016, the R-CNN family of algorithms dominated the field of target detection. After introducing YOLOv1, target detection algorithms have been differentiated into single-stage and two-stage. YOLO is characterized by abandoning the generation of candidate frames and adopting a direct regression approach for object classification and prediction. This dramatically simplifies the network structure and is nearly ten times faster than the detection speed of Faster R-CNN. As the YOLO family continues to grow, the current version of the YOLO framework has absorbed the advantages of the previous version. It is constantly innovating itself, with a broader range of applications in agriculture.

YOLOv8 is one of the latest YOLO architecture detectors, which inherits many of the advantages of real-time target detectors, including lightweight network architecture and powerful feature extraction capabilities with faster detection speed and higher detection accuracy. The Backbone part of YOLOv8 uses the CSPDarkNet network (Bochkovskiy et al., 2020), which applies a cross-stage hierarchical structure to the feature map merging, improving the accuracy and reducing the whole network’s computational complexity. YOLOv8 also borrows the ELAN structure from YOLOv7 (Wang et al., 2023) and designs the C2f module, which enriches the extracted feature information. YOLOv8 uses the CIoU (Zheng et al., 2021) to determine the IoU between prediction and ground-truth frames. In addition to that, its detection header separates the classification process and localization process, introduces Distributed Focus Loss (DFL) (Li et al., 2020), and also adopts the idea of Anchor Free, which eliminates the need for predefined anchors, making it more flexible and efficient compared to previous YOLO models. YOLOv8 provides models across various scales, including nano (n), small (s), medium (m), large (l), and extra-large (x). 

### 2.5 Improved YOLOv8s model

**Figure 3:**
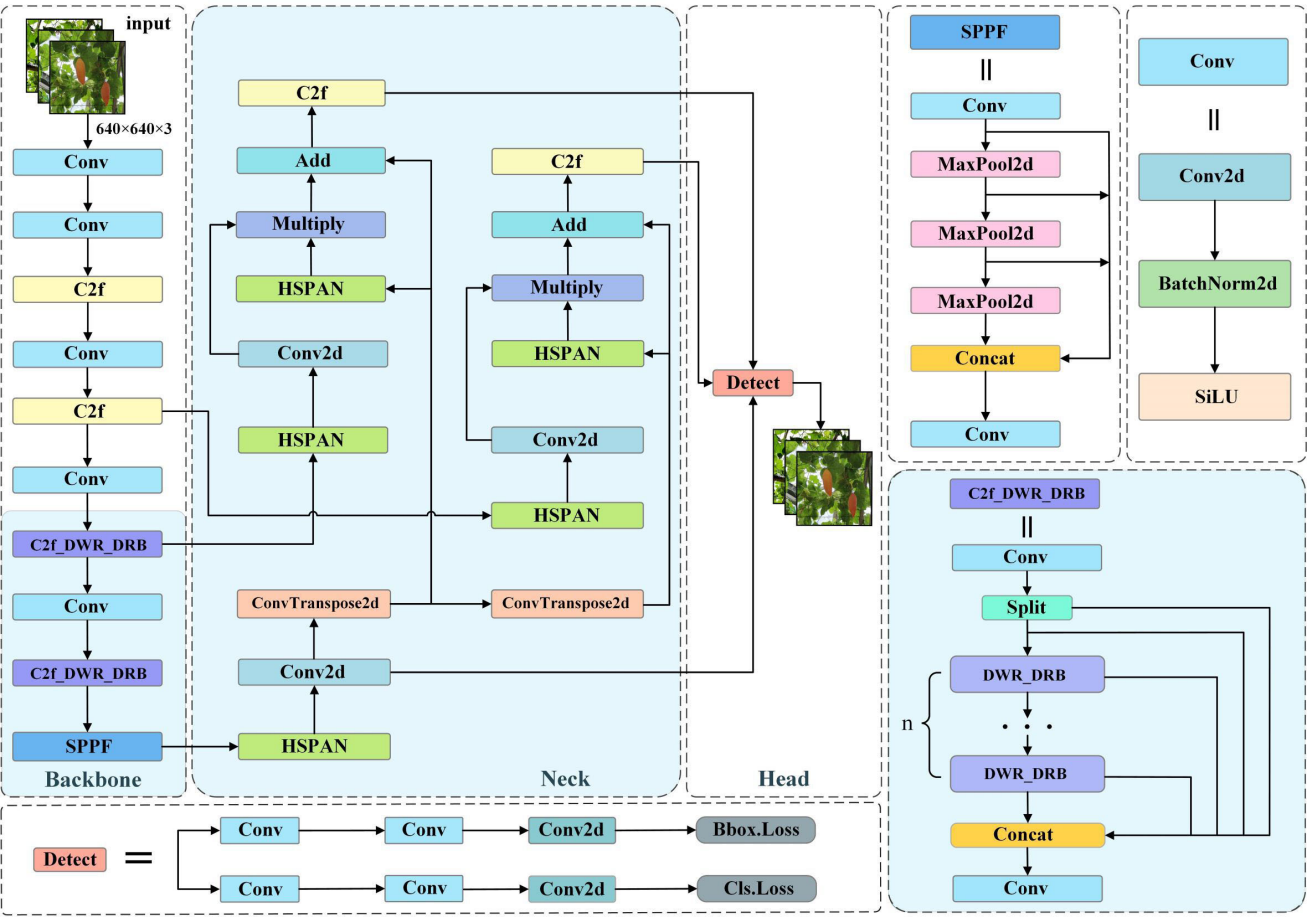
Improvement of YOLOv8 structure.

In this study, we considered the balance between computational cost and detection accuracy and chose YOLOv8s as the basic model. First, we designed the DWR-DRB module, which replaces the bottleneck of the original C2f module in Backbone to enhance the sensory field and constructed a new module, which we named C2f_DWR_DRB. Then, we constructed the HS-PAN architecture, which uses a bottom-up feature module to enhance the localization information. At the same time, it is combined with other layers in the Neck section to generate a more robust feature representation. The light blue background shows the central improvement part, and in the following two subsections, we describe the proposed method in detail. Our improved YOLOv8s model is shown in **Figure 3**.

### 2.6 Dilation-wise Residual-Dilated Reparam Block

In a dilated convolutional layer, a dilated convolutional layer utilizing a compact kernel is equated to a non-dilated (i.e., r=1 ) convolutional layer with a larger, sparser kernel, provided that disregarding specific input pixels is analogous to interspersing additional zeroes within the convolutional kernel. The original convolution kernel $\text{W}\in {ℛ}^{k\times k}$ becomes ${\text{W}}^{\prime }\in {ℛ}^{\left(\left(k-1\right)r+1\right)\times \left(\left(k-1\right)r+1\right)}$ after insertion of zeros, a process that can be realized by the transposed convolution of Equation (1) and the unitary kernel $\text{I}\in {ℛ}^{1\times 1}$ :


(1) $$\begin{array}{c} W^{\prime }=Conv_{transpose2d(W,I,stride=r).} \end{array}$$

Based on this equivalent conversion, DRB (Dilated Reparam Block) was proposed by Ding et al (Ding et al., 2023). in UniRepLKNet, which applies a solitary, unenlarged small kernel alongside several dilated small kernel layers to enhance a convolutional layer with a non-dilated large kernel.

By reparameterizing multiple blocks consisting of small kernel convolution layers with different dilated rates to be equivalently converted into a solitary large kernel convolution layer with a larger sparse kernel, DRB improves the detection network’s performance to extract spatial information while maintaining the number of learned Params and computational efficiency. This design innovation provides the convolutional network with a wider receptive field without increasing the depth of the model.

The essence of the DWR (Dilation-wise Residual) (Wei et al., 2022) module is a two-stage method for gathering information contextually across various scales, structured around a residual framework to capture nuanced details. The multi-scale sensory wild-formed feature maps are then fused, which reduces the difficulty of acquiring information. The first step is to generate relevant residual features based on the input features. The combination of 3×3 convolutional layers, BN layers, and ReLU layers generates many feature maps of different sizes as the material for the second step of morphological filtering. The second step is to perform morphological filtering on region features of different sizes. Initially, the region feature maps are segmented into several clusters, and then different groups are convolved in different ways.

We notice the similarity between the deep dilated convolution in the original DWR module and DRB. They both obtain a larger receptive field by improving the dilated convolution. Therefore, we utilize the method of reparameterizing and enhancing the non-dilated large kernel convolution layer in DRB to design the DWR-DRB module to replace the Bottleneck in C2f, which is utilized to gather information from various scales more efficiently, streamlining the process of contextual understanding. Specifically, we replace the deep convolution with dilated convolution of 3 in the second branch of the original DWR module with a DRB with a convolution kernel size of 5×5 , the deep convolution with dilated convolution of 5 in the third branch with a DRB with a convolution kernel size of 7×7 , and the 3×3 deep convolution in the first branch with dilated convolution of 0, and thus remains unchanged. In addition, the initial branch’s output channel was expanded to double the capacity compared to the subsequent branches due to the fact that more extensive spatial spanning connections require the help of more minor spanning connections.

**Figure 4:**
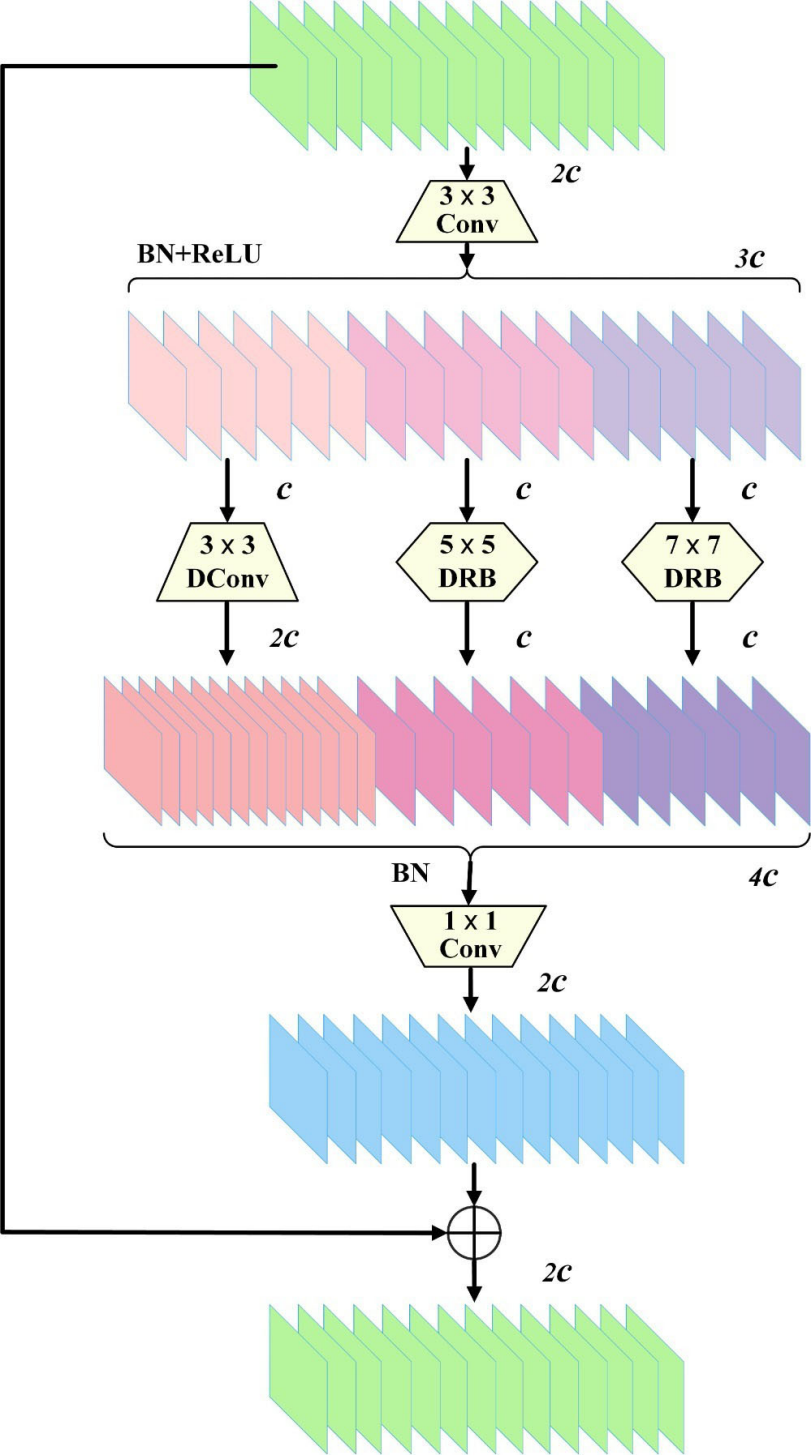
DWR-DRB (Dilation-wise Residual-Dilated Reparam Block) structure.

After plotting multi-scale contextual data, various results are consolidated to link all feature mappings. Features are then merged by batch normalization and point-by-point convolution and appended to the input feature map to build a more robust and holistic expression of the features. Schematic representation of the DWR module structure. **Figure 4** illustrates the three-branch DWR-DRB module of the high-level network structure. Conv denotes convolution, DConv denotes deep convolution, and c denotes the number of channels in a feature map.

### 2.7 High-level Screening-path Aggregation Networks

The color of the surface of immature fruits is close to the color of the surrounding leaves and canes, and coupled with the disruption caused by elements like fluctuating illumination and occlusion, the difference between Color-changing melons and the complex background becomes significant. To solve this problem, we refer to the multilevel feature fusion approach of HS-FPN (High-level Screening-feature Pyramid Networks) (Chen et al., 2024) and design the HS-PAN (High-level Screening-path Aggregation Networks) architecture for fusing multi-scale feature information to reduce the interference of complex backgrounds, thus improving the accuracy of fruit detection.

**Figure 5:**
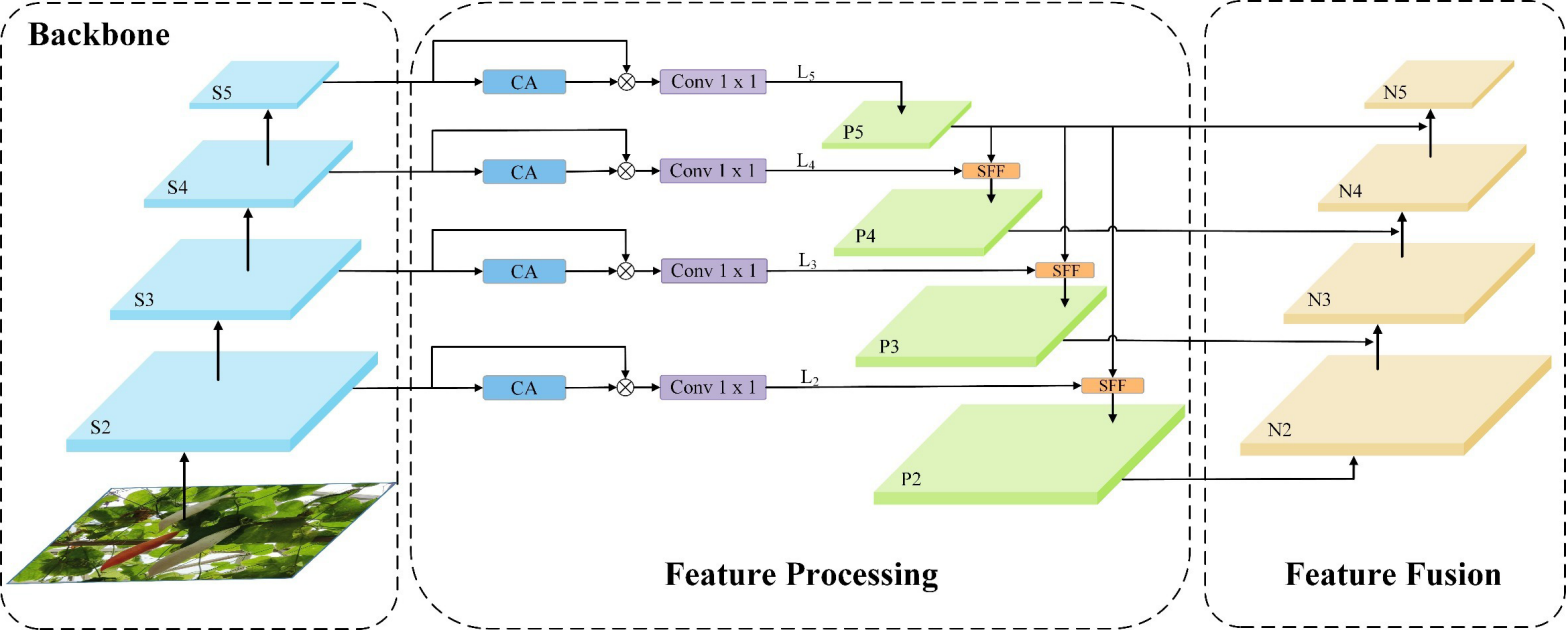
HS-PAN (High-level Screening-path Aggregation Networks) structure.

The structure of HS-PAN is shown in **Figure 5**. It consists of two sub-modules:(1) Feature processing module. (2) Feature fusion module. First, HS-FPN sieves through the feature maps derived from the Backbone at varying scales, subsequently amalgamating the information from upper and lower levels in the filtered feature maps using the Selective Feature Fusion (SFF) mechanism. Subsequently, in order to solve the drawback of FPN (Lin et al., 2017) in dealing with the ambiguity of high-level information, we constructed a bidirectional multilevel feature fusion PAN (Liu et al., 2018), i.e., HS-PAN, which adds a bottom-up feature fusion module, takes the low-level information as one of the input parts during feature fusion, and strengthens the localization information by taking advantage of the fact that the low-level features are more accurate for the target localization, which is the reason why we used the conventional C2f as a feature fusion mechanism in the beginning of the This is the reason why we use the regular C2f module when extracting features. This multilevel fusion approach has rich and comprehensive semantic information, which helps to obtain more detailed features in Color-changing melon images, thus enhancing the detection ability of the model.

We first introduce the Channel Attention (CA) and Dimensional Matching (DM) modules in the feature processing module. The CA module initially conducts global max and average pooling on the provided feature maps. This dual pooling strategy captures both the average and the critical features present. These are designed to filter out redundant information, compress features, and reduce the number of parameters. Combining the two pooling methods helps extract the critical information in each channel while ensuring minimal information loss. Next, the generated features are aggregated, and the Sigmoid function is employed to calculate the channel-wise weights in the network, which ultimately yields the weights for all channels. Subsequently, the weight information is multiplied by the feature maps of the corresponding scales to generate the filtered feature maps. The DM module adopts the point-by-point convolution method to match the feature maps of different scales and different numbers of channels before feature fusion and, at the same time, reduces the number of channels in each layer of the feature maps to 256. The SFF module, which is one of the core components of the HS-PAN, uses the high-level features as the filters to refine the low-level important information in the features to fuse multi-scale features more efficiently.

**Figure 6:**
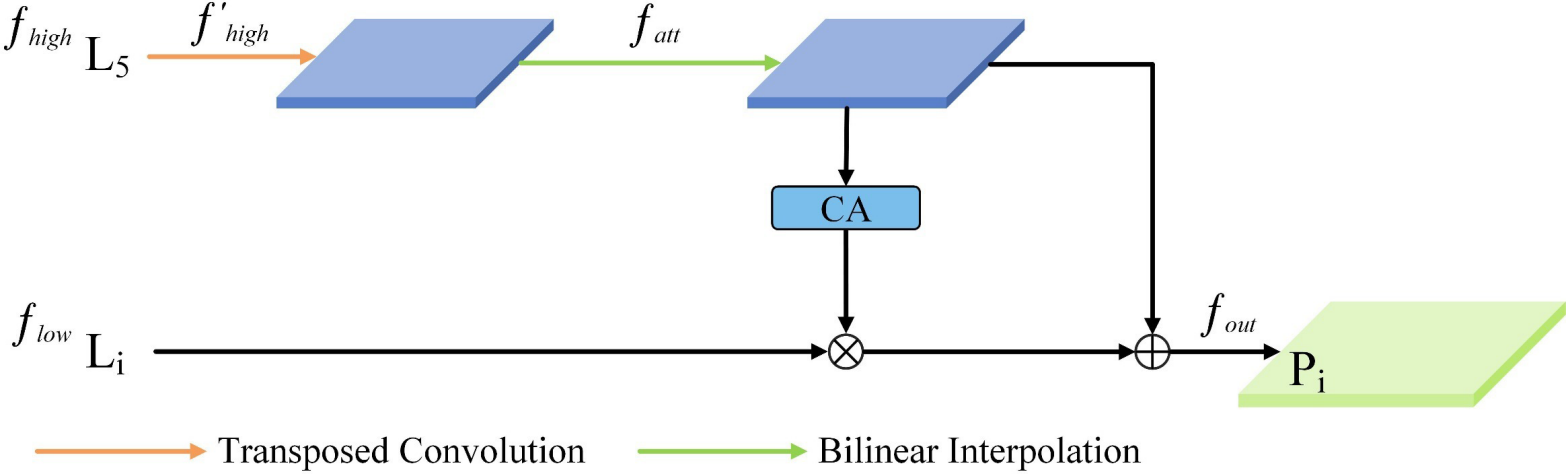
SFF (Selective Feature Fusion) module structure.


As illustrated in **Figure 6**, given a high-level feature $f_{high}\in R^{C\times H\times W}$ and a low-level feature $f_{low}\in R^{C\times H_{1}\times W_{1}}$ , where C denotes the channel count, H stands for the feature map’s height, and W stands for the feature map’s width. The high-level features are first dilated convolution, which is applied by a transposed convolution (T-Conv) with a 2-step and a 3×3 convolution to obtain the feature $f{'}_{high}\in R^{C\times 2H\times 2W}$ . Then, the high-level features are up-sampled or down-sampled using bilinear interpolation to align the dimensions of high-level and low-level features to obtain the feature $f_{att}\in R^{C\times H_{1}\times W_{1}}$ . Next, the CA module is utilized to unify the dimensionality of the attention weights generated from converting the high-level features and filtering the low-level features. Finally, the high-level features are fused with the filtered low-level features to obtain a more comprehensive feature $f_{out}\in R^{C\times H_{1}\times W_{1}}$ . Equations (2, 3) illustrate the process of feature fusion, where BL (Chen et al., 2018) is a multi-scale feature representation method.

(2) $$\begin{array}{c} f_{att}=BL(T-Con\upsilon \left(f_{high}\right)),\; \end{array}$$

(3) $$\begin{array}{c} f_{out}=f_{low}*CA\left(f_{att}\right)+f_{att}. \end{array}$$

### 2.8 Layer-Adaptive Sparsity Pruning

Model pruning productively decreases the number of model parameters and FLOPs (Lei et al., 2017). In neural networks, some parameters with relatively small weights take up a large amount of computational resources, but these redundant parameters have little effect on the results of model inference. By removing these parameters with smaller weights, the model can be compressed with little loss of accuracy, thus reducing memory consumption and alleviating computational load (Liu et al., 2017). Previous studies have found (Gale et al., 2019, Gale et al., 2020) that if the layered sparsity is chosen for a neural network, then a simple magnitude-based pruning (MP) can be a suitable balance between the model’s performance and lightweight. However, there is no clear solution to choosing the hierarchical sparsity.

**Figure 7:**
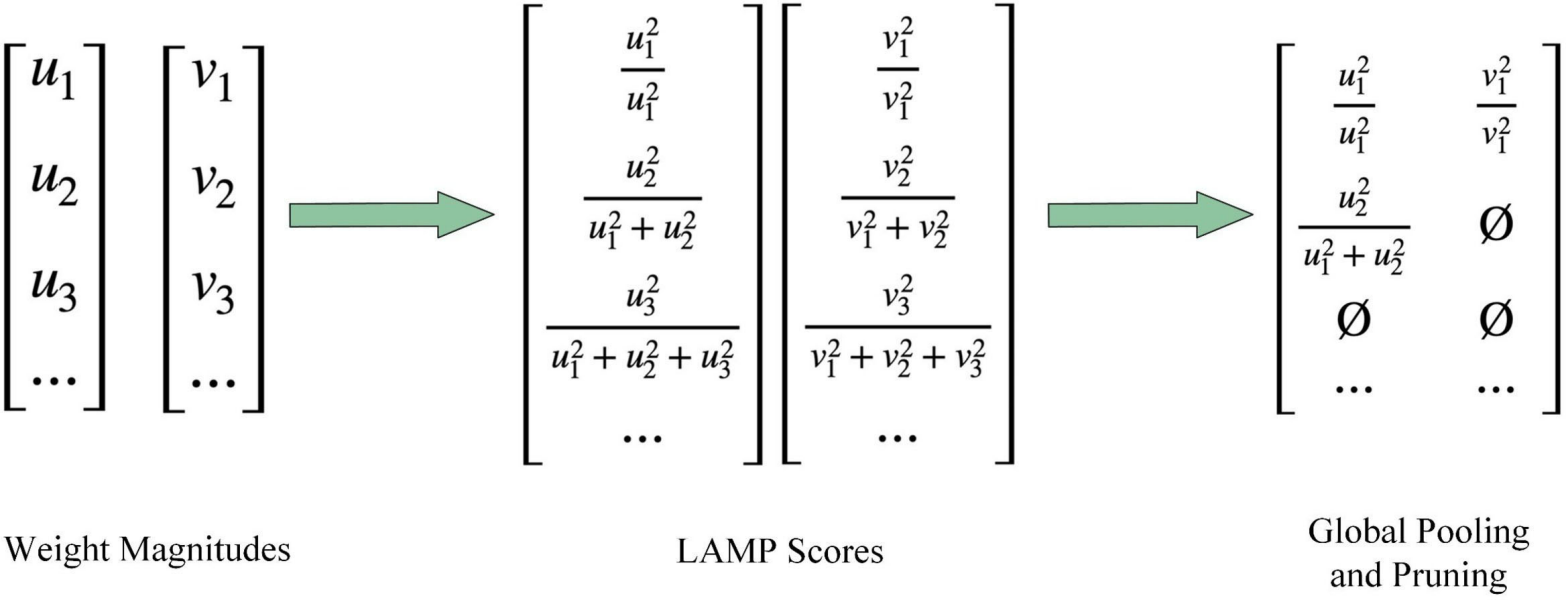
Illustration of the LAMP (Layer-Adaptive Sparsity Pruning) scores.

The model pruning method used in our research is the magnitude-based Layer-Adaptive Sparsity Pruning (LAMP) (Lee et al., 2020), which proposes a global pruning importance score. Global pruning is characterized by removing connections below the LAMP scores in the whole model rather than removing connections below a threshold score in each layer, i.e., global pruning is not equally sparse for each layer. As shown in **Figure 7**, the LAMP scores are the square of the weight size, normalized by the total of all remaining weights in the layer.

Consider a feedforward neural network of depth-d with corresponding weight tensors $W^{\left(1\right)},\ldots ,W^{\left(d\right)}$ for each convolutional layer and fully connected layer. Each weight tensor is assumed to be expanded into a one-dimensional vector to define the LAMP scores uniform for both the fully connected and convolutional layers. For these one-dimensional vectors, we assume the weights are sorted depending on the index map in ascending order, i.e., |W[u]|≤|W[v]| holds whenever u<v , where W[u] denotes the entries of W that are mapped by the index u . The LAMP scores corresponding to the u -th position in the weight tensor W are established in the Equation (4).


(4) $$\begin{array}{c} score\left(u;W\right):=\frac{{\left(W\left[u\right]\right)}^{2}}{\sum_{v\geq u}{\left(W\left[u\right]\right)}^{2}} \end{array}.$$

Once the LAMP scores are computed, the minimum-scoring connections are globally pruned until the required global sparsity constraints are satisfied. That is, for any given weight tensor W , along with indicators u and v , the following conditions need to be satisfied in the Equation (5).


(5) $$\begin{array}{c} {\left(W\left[u\right]\right)}^{2}>{\left(W\left[v\right]\right)}^{2}\Rightarrow score\left(u;W\right)>score\left(v;W\right) \end{array}$$

**Figure 8:**
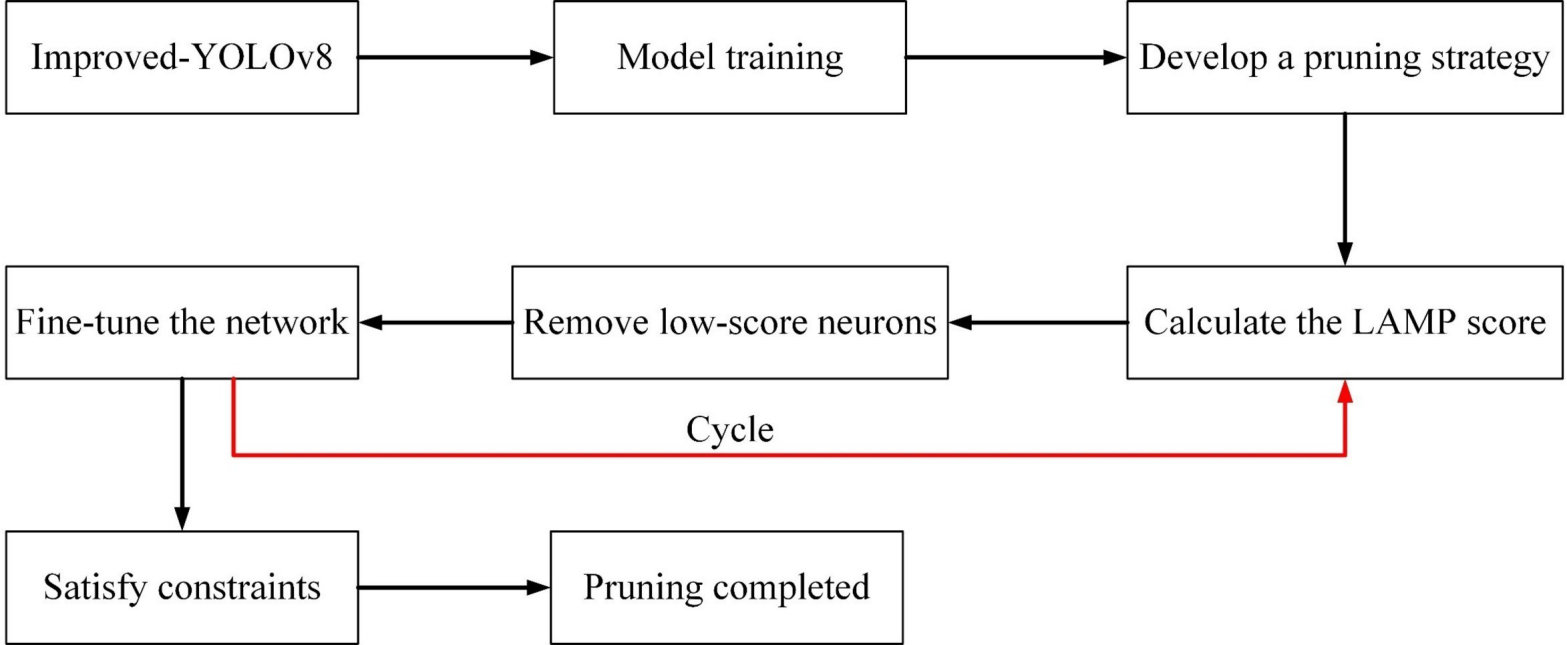
Model pruning flowchart.

All connections with the LAMP scores less than the target weights are pruned. Global pruning using the LAMP scores is similar to MP-based hierarchical pruning with automatic selection of hierarchical sparsity. Pruning the model with the LAMP scores after training maintains the benefits of MP, and the LAMP scores do not depend on any model-specific knowledge, eliminating the need for a long, sparse training step. The overall process of pruning is depicted in **Figure 8**.

### 2.9 Block-Correlation Knowledge Distillation

Knowledge distillation is a classical approach to model compression, a concept initially put forward by Hinton et al (Hinton et al., 2015), and during the following years, researchers have proposed many more knowledge distillation methods, such as Logits distillation and Features distillation (Adriana et al., 2015; Ahn et al., 2019; Heo et al., 2019). The core idea of knowledge distillation is to extract knowledge from the better-performing and more complex structure of the teacher model and transfer the knowledge to the more lightweight student model without changing the network structure so that the performance and versatility of the smaller model can be enhanced.

Previous approaches obtained good results but performed poorly on small datasets. Therefore, we adopted BCKD (Wang et al., 2023). Unlike conventional Logits distillation or Features distillation, BCKD notices the connections between blocks in a neural network, providing new knowledge for distillation. This approach improves performance, does not introduce additional computational overhead, and addresses the problem of poor distillation on small datasets.

**Figure 9:**
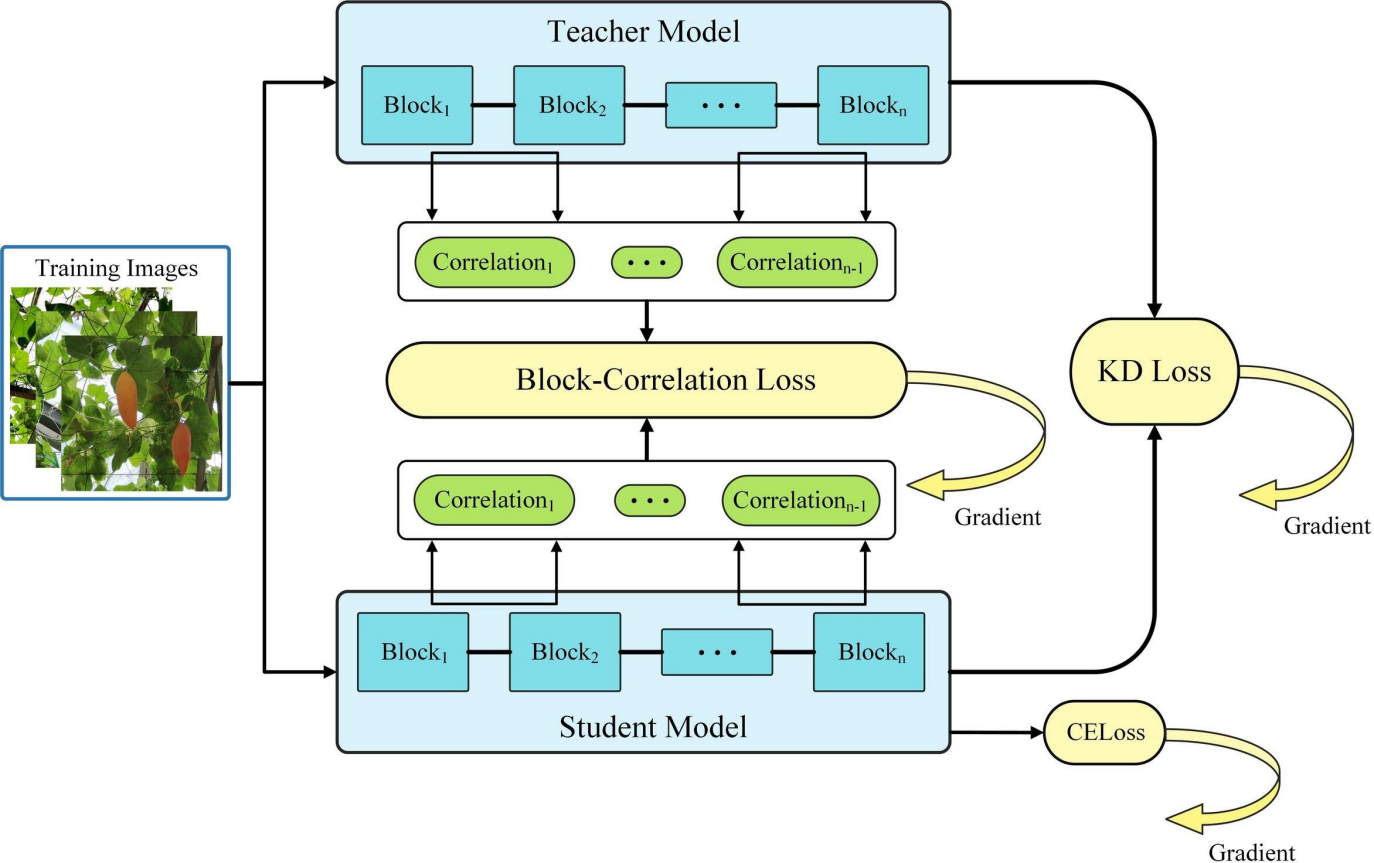
BCKD (Block-Correlation Knowledge Distillation) structure.

**Figure 9** illustrates the structure of the BCKD. The classification task’s bottom right corner is the cross-entropy loss function (CE). The bottom uses conventional knowledge distillation (KD) for the trained student model. Finally, the block correlation loss function (BC) is employed to augment the distillation’s efficacy.

BCKD uses ResNet32x4 and ResNet8x4 (He et al., 2016) as infrastructure. Setting the set $\text{B }=\left\{b_{1},\ldots ,b_{i},b_{n}\right\}$ be the output candidates for each residual block from teachers and students, and the correlation between neighboring blocks is assumed to be a relationship that the model can learn, denoted as $\text{C }=\left\{c_{1},\ldots ,c_{j},c_{n-1}\right\}$ . All candidate outputs have their own feature mapping size and channel dimension, e.g., $b\in {\mathbb{R}}^{B_{s}\times D\times H\times W}$ , where $B_{s}$ 、 D 、 H and W denote the batch size, channel dimension, height, and width, respectively. When the number of neighboring blocks is n , it is clear that the number of correlations is n−1 . For ease of exposition, we use $b_{s},b_{t},c_{s}$ and $c_{t}$ to denote the blocks and correlations of students and teachers, respectively. From this, we derive the equation for the correlation set C :


(6) $$\begin{array}{c} \hat{b_{\text{i}-1}}=\psi^{D}\left(adaptive_{avg\left(b_{\text{i}-1}\right)}\right). \end{array}$$

(7) $$\begin{array}{c} c_{\text{j}}=\phi \;\left(\hat{b_{\text{i}-1}}⊙{\hat{b_{\text{i}}}}^{\text{T}}\right)\;\;\;\;\;\;\hat{c_{\text{j}}}=\phi \;\left(\hat{b_{\text{i}}}⊙{\hat{b_{\text{i}-1}}}^{\text{T}}\right) \end{array}$$

The adaptive_avg(·) in Equation (6) is a convolutional kernel self-adaptive function that is used to average the values of the spatial dimensions of the feature maps, whereby $b_{\text{i}-1}$ can be unified according to the size of the feature maps of $b_{\text{i}}$ . $\psi^{D}\left(\cdot \right)\;$ represents the average pooling of the channels, so the feature map sizes of $\left\{\hat{b_{\text{i}-1}},\hat{b_{\text{i}}}\right\}\in {\mathbb{R}}^{B_{s}\times H_{i}\times W_{i}}$ have feature mappings of equal size. The ϕ(·) in Equation (7) represents the normalized softmax function, thus $\begin{array}{c} c_{\text{j}}\in {\mathbb{R}}^{B_{s}\times H_{i}\times H_{i}} \end{array}$ and $\hat{c_{\text{j}}}\in {\mathbb{R}}^{B_{s}\times W_{i}\times W_{i}}$ .

Next, we utilize the multilayer perceptron (MLP) to help match $b_{s}$ and $c_{s}$ with $b_{t}$ and $c_{t}$ in order to preserve the features while trying to apply different network structures. Considering the important influence of the discriminative classifier on the model’s detection capability, we input the results obtained from the MLPs into the classifiers of the teacher’s model with the aim of obtaining a more comprehensive representation of the correlation features. The resultant $MLP_{out}$ and $CLS_{out}$ are expressed are expressed in the Equations (8), (9).


(8) $$\begin{array}{c} MLP_{out}=L2_{Norm\;}\left(Linear\left(ReLu\left(Linear\left(\bar{x}\right)\right)\right)\right)\;, \end{array}$$

(9) $$\begin{array}{c} CLS_{out}=Cls(MLP_{out}). \end{array}$$

Where Linear(·) denotes the linear layer, ReLu(·) denotes the ReLu activation function, $L2_{Norm}\left(\cdot \right)$ stands for L2 for normalization, Cls(·) denotes the classifier of the teacher model, $\bar{x}$ denotes $c_{\text{j}}$ or $\hat{c_{\text{j}}}$ . In $MLP_{out}\in {\mathbb{R}}^{C\times B_{s}\times M}$ and $CLS_{out}\in {\mathbb{R}}^{C\times B_{s}\times L}$ , C is the number of elements in the set C , $B_{s}$ is the batch size, and M and L stand for the output dimensions of $MLP_{out}$ and $CLS_{out}$ respectively.

After the above process, the correlations of the student model and the teacher model are then mapped into the same feature space. In this feature space, the correlation between neighboring blocks of the pre-trained teacher model is strong, and the distribution of different samples is consistent. In contrast, the untrained student model is divergent across samples. Therefore, for the student model to understand the gap between itself and the teacher $MLP_{out}$ and $CLS_{out}$ , it can learn more knowledge and thus improve the performance of the student itself.

Regarding loss function, if L1 (MAE) or L2 (MSE) is used directly, it does not work well for the untrained student model. The MSE loss function performs better for the model in terms of gradient and convergence, and the MAE loss function performs more consistently when dealing with outliers. Therefore, BCKD chose Huber loss (Gokcesu and Gokcesu, 2021) to combine the respective advantages of MAE and MSE in order to get better performance from the student model.

### 2.10 Pseudo code for combinatorial algorithms

By integrating the approaches in the above subsections, we designed an algorithm for Color-changing melon maturity detection, divided into five main steps: initialization, model training, model pruning, knowledge distillation, and testing. The algorithm enhances the robustness of the model when dealing with complex scenarios while maintaining the lightweight characteristics of the model, and the pseudo-code is given in **Algorithm 1**.

Algorithm 1 **Pseudo code for combinatorial algorithms.**

**Input:** Color-changing melon images, pretrained YOLOv8s weight
**Output:** Category confidence and prediction frame coordinates
1: **Initialize:** img_split=img_train(80%) + img_val(80%) + img_test(80%);
2: **Training on Server:**
3: batch_size=64, img_size=640, epochs E_t_= 300;
4: **for** *i* = 1: *E_t_
* **do**
5:  Train on the img_train with the improved YOLOv8;
6:  Calculate the loss function;
7:  Evaluate model using img_val;
8: **end for**
9: save best_weight.pt;
10: **Model Prune on Server**
11: model=best_weight.pt, epochs E_p_= 9999, pruned_method=LAMP, speed_up=3.0;
12: **for** *i* = 1: *E_p_
* **do**
13:  if(speed_up > 3.0)
14:  break;
15:  Calculate the LAMP score for each channel in the improved model;
16:  Remove the connection with the minimum score and calculate speed_up;
17: **end for**
18: save last_prune.pt;
19: **Knowledge Distillation on Server**
20: model= last_prune.pt, epochs E_kd_= 250, kd_method=BCKD, teacher=YOLOv8s-Improved;
21: **for** *i* = 1: *E_kd_
* **do**
22:  Predict training data and generate sample distribution space;;
23:  Utilize BCKD to help students calculate the difference between their own and their teacher’s predictions of outcomes;
24:  Update the parameters of the student model using the loss function;
25: **end for**
26: save best_kd_weight.pt;
27: **Testing on laptop**
28: Predict model using img_test;
29: Obtain the output result.

## 3 Results and discussion

### 3.1 Experimental setup

This study’s experiments were all performed on an Ubuntu 18.04 system; the programming language was Python 3.9.16, and the network framework used was Pytorch 1.10.0 (cuda 11.7). For our training phase, we used a high-performance server configured with an Intel^®^ i7 13700K 16C5.40GHz CPU and an NVIDIA RTX 4090 GPU. For the inference testing phase, we used an Intel^®^ i7 8750H 4C2.20GHz and an NVIDIA GTX 1050ti laptop to simulate a resource-constrained device and test the model’s performance. The hyperparameter settings for the training phase include a batch size of 64 and 300 epochs for the number of training rounds, and the officially provided pre-training weights are used as the initial weights. The rest of the hyperparameters are the default values of YOLOv8.

### 3.2 Evaluation indicators

In this study, a total of seven metrics, namely, precision, recall, average precision, model size, number of Params, FLOPs, and FPS, are used to comprehensively evaluate the performance of the model.

The formulas for precision and recall are expressed in the Equations (10), (11).


(10) $$\begin{array}{c} \text{Precision}=\frac{TP}{TP+FP}, \end{array}$$

(11) $$\begin{array}{c} \text{Recall}=\frac{TP}{TP+FN}. \end{array}$$

In the above two equations, TP stands for the number of targets correctly judged as positive, FP stands for the number of targets incorrectly judged as positive, and FN stands for the number of targets belonging to positive but incorrectly judged as negative. AP is used to calculate the average precision of a single class of targets under different recall rates, and mAP is used to calculate the average AP of multiple classes of targets, and their definitions are expressed in the Equations (12), (13), respectively:


(12) $$\begin{array}{c} AP=\int_{0}^{1}P\left(R\right)dR, \end{array}$$

(13) $$\begin{array}{c} mAP=\frac{1}{N}\sum_{i=1}^{N}AP. \end{array}$$

For all kinds of targets detected, the higher the mAP , the more accurate the model’s predictions are; therefore, it represents a better detection performance of the model. mAP@0.5 denotes the average AP of each kind of target when the IoU threshold is set to 0.5. mAP@0.5:0.95 The thresholds are computed from the range of 0.5 to 0.95, with an increase of 0.05 in each step, and the obtained average AP of each kind of the average AP of the targets, they are defined in the Equations (14), (15), respectively:


(14) $$\begin{array}{c} mAP@0.5=\frac{1}{N}\sum_{i=1}^{N}AP_{i}. \end{array}$$

(15) $$\begin{array}{c} mAP@0.5:0.95=\frac{1}{N}\sum_{i=1}^{N}\sum_{j}AP_{i}\;\left(j=0.5,0.55,0.6,\ldots ,0.95\right). \end{array}$$

Params denote the sum of parameters to be trained within the model. FLOPs denote the number of floating point operations required during network training, and model size denotes the size of the model. The lower these three metrics are, the more lightweight the model is and, therefore, the more suitable it is for deployment on edge devices.

Frames per second ( FPS ) measure the model’s detection speed. FPS is calculated from the inverse sum of the pre-processing time, inference time, and post-processing time. The larger its value, the faster the real-time detection of the model. Its definition is in the Equation (16).


(16) $$\begin{array}{c} FPS=\frac{1}{t_{pre-process}+t_{\text{inf}erence}+t_{post-processing}}. \end{array}$$

### 3.3 Comparison of before and after improvements

**Figure 10:**
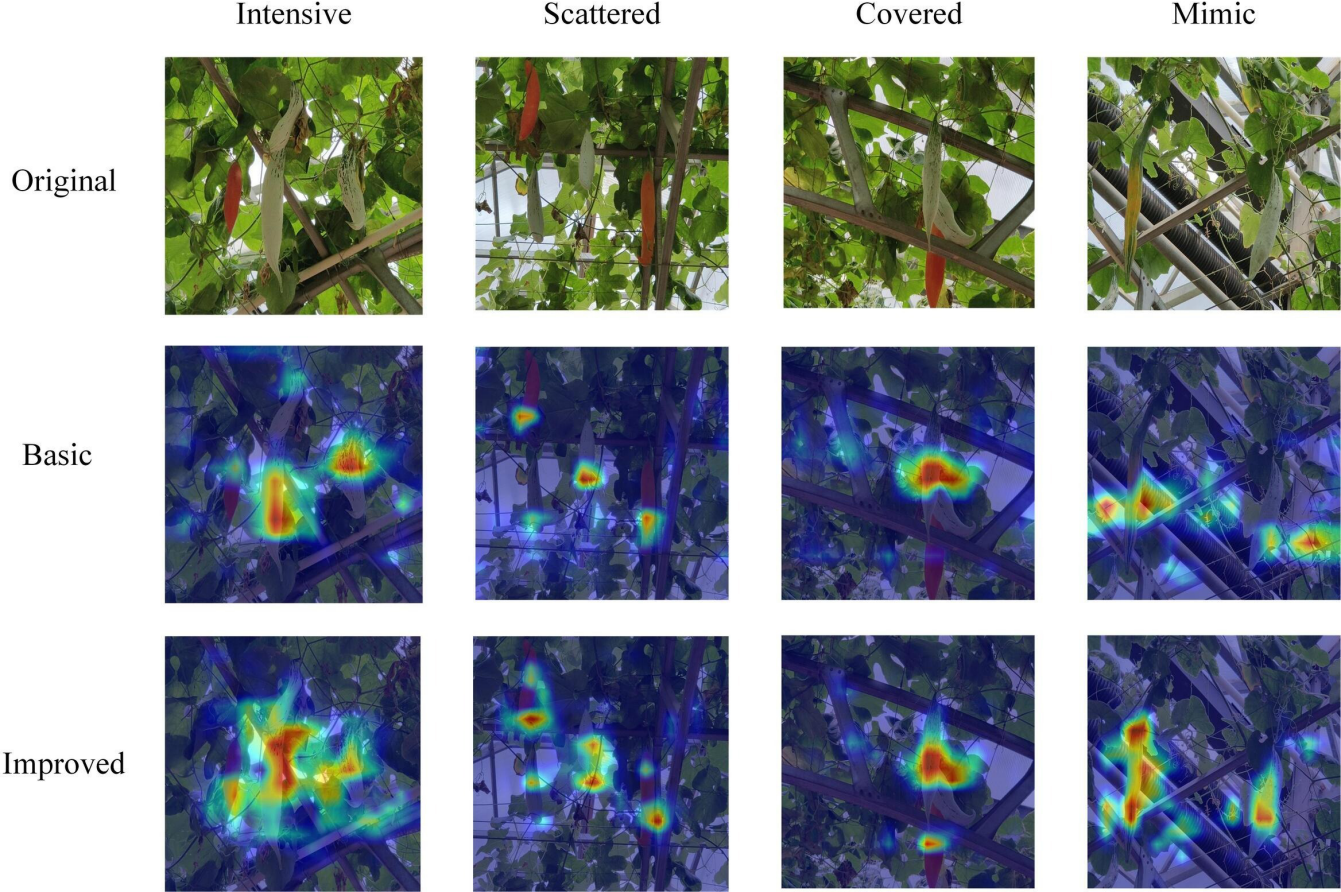
Comparison of visualized heat maps in different scenarios.

We trained the base YOLOv8s and the improved YOLOv8 separately with the same parameter settings on the server side and then compared the hotspots of attention of the two models under four scenarios on the test set on the laptop side. As shown in **Figure 10**, when the fruits are denser, the improved model shows a higher degree of hotness for the fruit-concentrated regions, while the hotspots of the base model are more scattered. When the fruits are more dispersed, the improved model has more hotspots and a little more heat for the regions where the fruits are located. For regions where the fruits overlap, the improved model pays more attention to the occluded fruits, while the base model pays less attention to the occluded fruits. The final image shows the similarity between the background and the fruit, which is similar to the common mimicry in the insect world, and it can be seen that the base model mistook the white pipe on the right side for an immature fruit while the improved model shows the hotspots of attention to the fruit very well.

**Figure 11:**
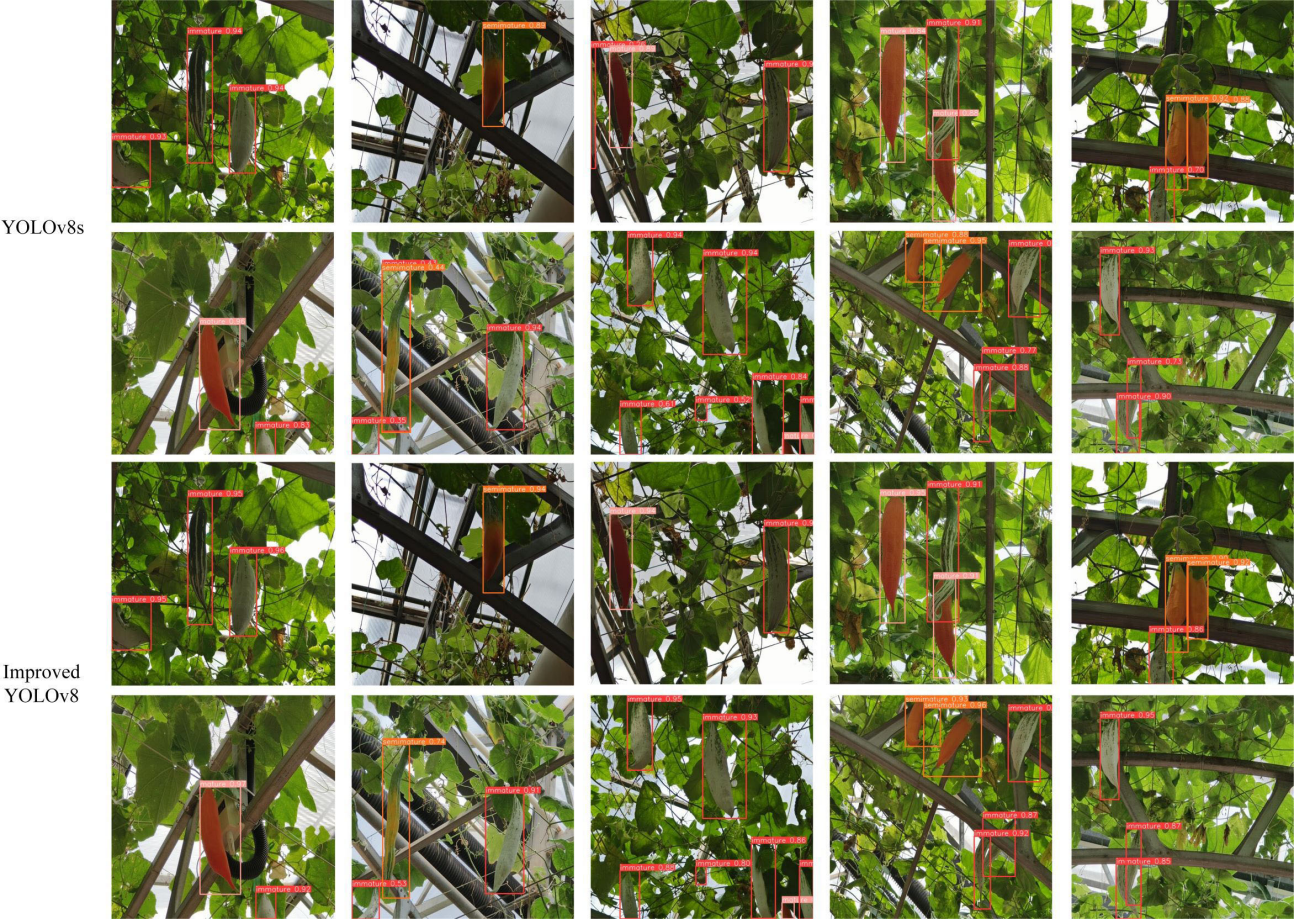
Comparison of detection effect.

**Figure 11** shows some of the detection results of the two models on the test set. 

In the first row of Color-changing melon detection, we list the detection effects of the two models when the usual scene, backlight, and fruit overlap, respectively. Both models perform better, but the confidence of the improved model is generally higher than that of YOLOv8s, and there are no misdetected fruits. When facing smaller fruits and fruits at the edge of the figure, the improved model has better detection performance, and the confidence level is 0.28 higher than that of YOLOv8s. When switching to the second row of Color-changing melon detection, we compared the detection effects of the two models when the fruits are at the edge of the figure, dense fruits, and fruits are obscured by other objects, and the confidence level of the improved model is still higher than that of YOLOv8s, especially when other objects obscure the fruits. They are more effective when other objects occlude them. However, given the rigor of the article, we also give examples of rare errors in the detection process of both models, as the co-obscuration of leaves and other objects creates a visual misalignment, which results in a situation where both models detect the same fruit as two targets.

Overall, YOLOv8s misdetected the background as fruit in a few cases and had problems with ambiguous judgments about the maturity of some fruits. In contrast, the improved model is more accurate in localizing and classifying fruits with a higher confidence level.

**Figure 12:**
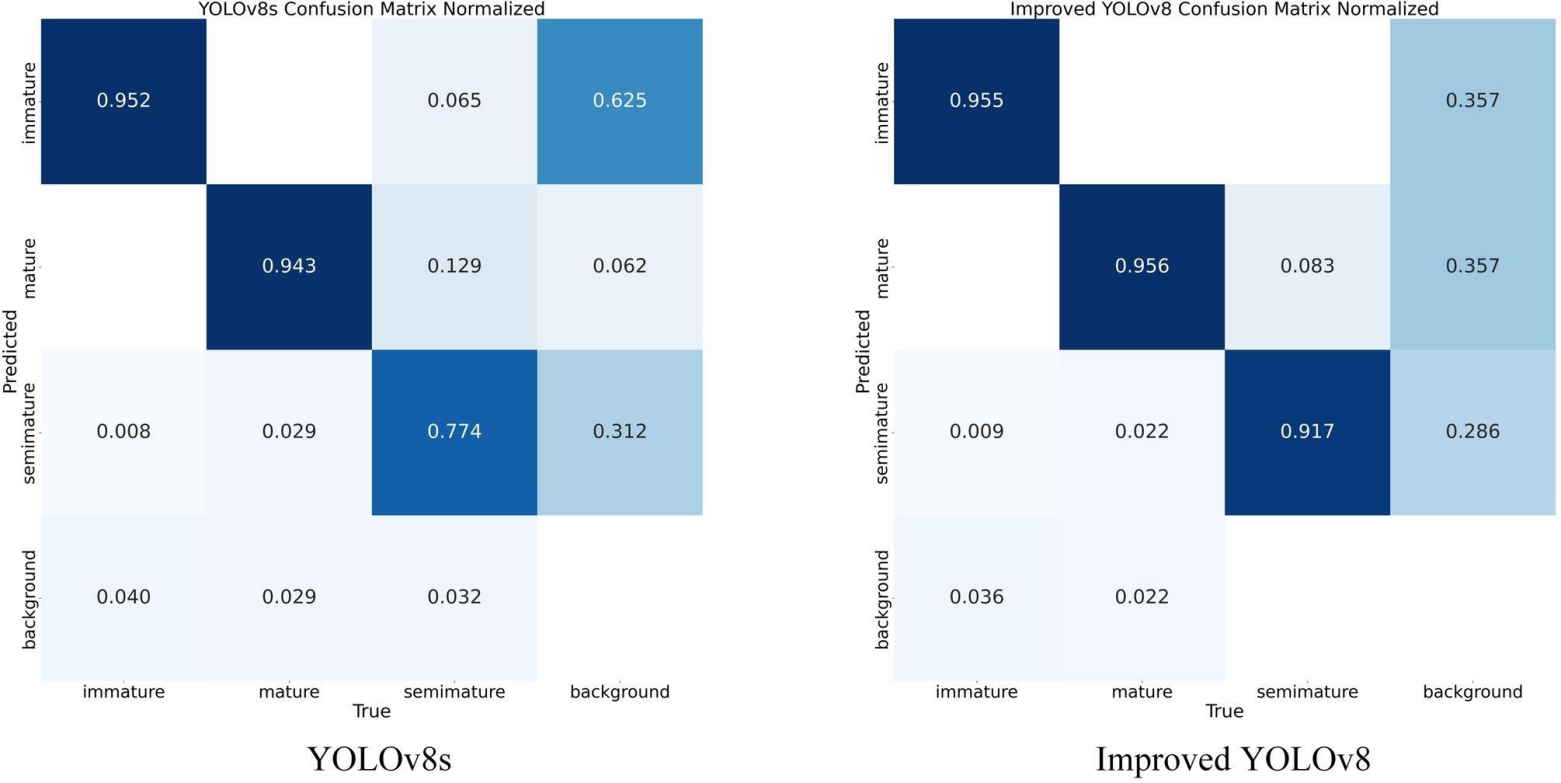
Comparison of confusion matrices.

In order to illustrate more intuitively the prediction accuracy of the three categories of fruit maturity before and after the model improvement, we present the normalized confusion matrix of the training results. As depicted in **Figure 12**, in the confusion matrix, the rows indicate the predicted labels for the categories, and the columns indicate the true labels for the categories. In each square, darker colors indicate larger values, lighter colors indicate lower values, and white indicates empty values. By looking at the differences between the predicted values for each category, it can be observed that the values of the improved model’s diagonal lines add up to a larger sum and improve the accuracy of the predictions for semi-mature fruits, as well as reduce the proportion of backgrounds that are mistakenly detected as immature fruits. This demonstrates that the multilevel feature fusion mechanism we constructed reduces the interference of complex background on fruit detection accuracy to some extent.

### 3.4 Results of ablation experiments

The server’s ablation experimental results are recorded in **Table 1**. The first row uses YOLOv8s as the baseline, and each module can be added to the model independently. Where A denotes the use of the DWR-DRB module, B stands for the use of the HS-PAN module, and A+B denotes the merging of the two model optimization methods into the base model. As seen from the table, each module suggested in this research contributes to the model performance when merged into the base model and reduces the network structure’s complexity and the associated computational workload.

**Table 1:** Results of ablation experiments.

A	B	A + B	mAP@0.5	mAP@0.5:0.95	Precision	Recall	Params	GFLOPs
			94.8%	86.5%	98.6%	97%	11.13 M	28.4
√			95.0%	88.6%	97.7%	98%	10.46 M	27.4
	√		96.2%	87.1%	96.6%	99%	7.73 M	25.0
		√	95.5%	89.0%	98.3%	99%	6.47 M	22.8

The "√" symbol indicates that the module is used.

### 3.5 Results after model pruning

**Figure 13:**
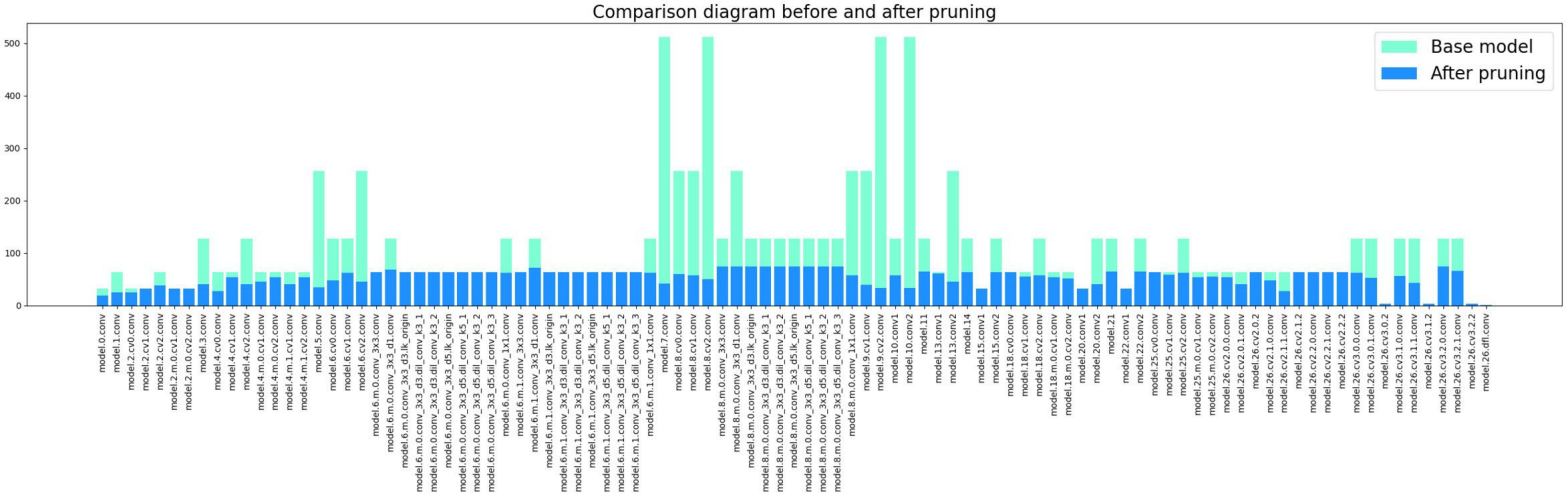
Comparison before and after channel compression.

After several experiments, we adopted a pruning acceleration ratio of 3.0 for the improved model because the pruning rate at this point can make the mAP stay relatively good. During the neural network’s pruning phase, the layer with the high LAMP scores has a higher importance level of its channels. Thus, a small amount of pruning or directly skipping the pruning of the channels of that layer can be done to remove the non-essential connections more reasonably to maintain the performance of the pruned model. **Figure 13** demonstrates the changes in the number of channels in each layer of the pruned model. It can be seen that some of the convolutional layers are pruned strongly, while the float in the number of channels in most of the layers is not significant. After pruning, the range of channel counts changed from 1 to 512 before pruning to 1 to 74 after pruning. mAP 0.5 and mAP@0.5:0.95 were reduced by 0.8% and 2.9%, respectively. The number of model Params decreased from 6.47 M to 1.14 M, a reduction of 82%. The number of computed FLOPs is reduced from 22.8 GFLOPs to 7.5 GFLOPs, a 67% reduction. The model size is also changed from 12.64 MB to 2.47 MB.

### 3.6 Effect of different teacher models on distillation

**Figure 14:**
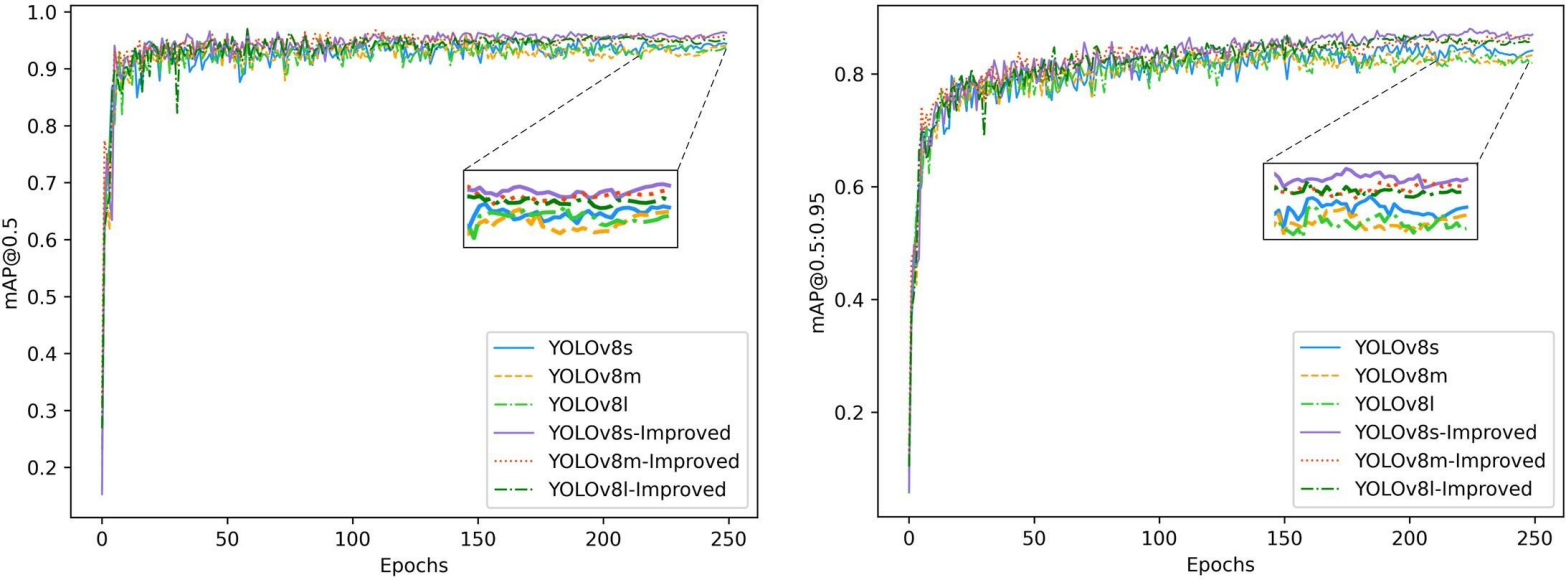
Validation results of the teacher model at different scales.

After a model is pruned, the accuracy generally decreases by a certain degree, and it is generally essential to adjust the pruned model accordingly with the aim of restoring its accuracy. Since the structure of the pruned model is relatively simple, it has good portability while ensuring prediction accuracy when the task goal is relatively clear. We distill the pruned model as a student model using the BCKD method. To evaluate the impact of various size scales of teachers on the effect of the student models, we trained the students on their knowledge using the base YOLOv8 and the improved model in s, m, and l sizes, respectively. **Figure 14** illustrates the validation results on a laptop after distillation training with different teacher models. **Table 2** provides specific data after validation of the distillation models. I-YOLOv8 in the table represents the improved YOLOv8 model.

**Table 2:** Validation results of distillation by different teachers.

Teacher-model	mAP@0.5	mAP@0.5:0.95	Precision	Recall	FPS
YOLOv8s	95.5%	86.1%	56.2%	99%	63.8
YOLOv8m	95.1%	85.9%	57.4%	99%	63.8
YOLOv8l	94.0%	84.6%	58.9%	99%	63.6
I-YOLOv8s	96.1%	88.1%	97.8%	99%	78.9
I-YOLOv8m	95.6%	86.6%	95.3%	99%	78.7
I-YOLOv8l	95.3%	86.3%	95.9%	99%	78.4

According to the comparison results in the table, it is easy to see that the base model of different sizes is not as effective in training the students as the improved model. Furthermore, the distillation of the student model by the untrained base model is too low in accuracy, and there is a cliff drop in detection speed. While the teacher models themselves continue to improve, they perform less and less well for distillation training. Compared to the other models as teachers, Improved-YOLOv8s had the most outstanding results for student training when the student model achieved 96.1% mAP 0.5 and 88.1% mAP@0.5:0.95. YOLOv8l had the worst distillation training as a teacher when the student model achieved 94.1% mAP0.5 and 88.1% mAP 0.5 only 94.1% and mAP@0.5:0.95 only 84.6%. This indicates that when the gap between the teacher and student models is within a reasonable range, the student model obtains more effective knowledge from the teacher network. On the contrary, when the gap between the two is too large, the student model is unable to learn much of the knowledge from the teacher’s network, and the training effect is not as good as expected. Therefore, we choose the s version of the improved model as the teacher network to distill the pruned model.

### 3.7 Comparison between different target detection networks

To evaluate the efficacy of our suggested approach, we trained various lightweight networks in the same server-side experimental environment. We tested the trained models on a laptop to simulate the environment of resource-constrained hardware. The specific experimental results are shown in **Table 3**. In the table, I-YOLOv8 represents the improved YOLOv8 model, I-P-YOLOv8 represents the improved model trained with fine-tuning after pruning, and I-P-KD-YOLOv8 represents the improved model trained with knowledge distillation after pruning. Under the condition of mAP@0.5, I-YOLOv8 and I-P-KD-YOLOv8 showed better results compared to other models. When the evaluation metric becomes the more stringent mAP@0.5:0.95, the models with a larger number of Params and computed FLOPs take advantage, leading the other lightweight networks by 5% to 10%, which can be seen in the good performance of YOLOv8s as well as our subsequent improved models, and among them I-YOLOv8 is also 2.5% higher than YOLOv8s. In terms of precision and recall, the performance of the various lightweight networks does not differ much. After pruning, the FLOPs and Params of I-P-YOLOv8 are significantly lower than other lightweight networks and even smaller than YOLOv8n. Meanwhile, the detection speed of I-P-YOLOv8 is somewhat improved, which is 9.1% and 14% faster than YOLOv8s and I-YOLOv8, respectively. Subsequently, after knowledge distillation, I-P-KD-YOLOv8 shows a large improvement in the metrics of mAP, and the overall performance outperforms that of I-P-YOLOv8. It can be seen that I-P-KD-YOLOv8 maintains the detection performance of I-YOLOv8. Meanwhile, the number of parameters and FLOPs are significantly reduced, and the model size is the smallest, which is an excellent balance between accuracy, speed, and model lightweight. YOLOv8s has higher detection accuracy than YOLOv8n, while the parameters and computational effort are much smaller than YOLOv8m. S-scale models can strike a good balance between detection performance and model complexity relative to n- and m-scale YOLOv8 models, so we chose YOLOv8s as the original model and improved it. After subsequent model pruning and knowledge distillation operations, the detection accuracy of the improved YOLOv8s is higher than that of YOLOv8n. However, it is more lightweight, making it more suitable for robot picking for automated Color-changing melons.

**Table 3:** Comparison of different target detection networks.

Model	mAP@0.5	mAP@0.5:0.95	Precision	Recall	Params	GFLOPs	FPS	Size
YOLOv4-tiny	94.4%	74.5%	93.8%	93%	5.88 M	16.2	52.0	22.4 MB
YOLOv5s	93.5%	78.6%	93.8%	96%	7.03 M	16.0	55.1	14.5 MB
YOLOv7-tiny	93.4%	70.8%	94.0%	99%	6.01 M	13.1	66.5	12.3 MB
YOLOv8n	94.1%	81.2%	95.7%	99%	3.01 M	8.1	64.8	6.2 MB
YOLOv8s	94.8%	86.5%	98.6%	97%	11.13 M	28.4	72.3	21.5 MB
I-YOLOv8	95.5%	89.0%	98.3%	99%	6.47 M	22.8	69.2	12.6 MB
I-P-YOLOv8	94.7%	86.1%	96.0%	99%	1.14M	7.5	78.9	2.5 MB
I-P-KD-YOLOv8	96.1%	88.1%	97.8%	99%	1.14M	7.5	78.9	2.5 MB

## 4 Conclusion

In this study, we focus on the need for robotic picking of Color-changing melons and put the front-loaded fruit detection work into the study, which lays the foundation for further realization of automatic picking work. We first designed the DWR module and DRB to expand the receptive field, aiming to further strengthen the Backbone part’s ability to acquire multi-scale contextual information. Subsequently, we design HS-PAN with multi-level feature fusion to strengthen the localization information and enrich the semantic information in the feature fusion process, which helps to enhance the detection network’s attention to Color-changing melons’ details, thus increasing the accuracy of fruit detection and reducing the proportion of false detections. Then, we simplified the improved network structure by pruning unimportant connections in the detection network using Layer-Adaptive Sparsity Pruning. Finally, the accuracy of the pruning model is further recovered using Block-Correlation Knowledge Distillation and compared to other lightweight networks. To summarize, we first improve and train modules on larger models for complex scenarios. Next, we drastically simplify the network structure of the improved model by model pruning. Finally, we make the accuracy of the pruned model close to the pre-pruning level by knowledge distillation. The advantages of our proposed combinatorial algorithm are that it obtains higher accuracy and stronger robustness than other lightweight models. In contrast, the complexity of the final model is much lower than that of the lightweight networks. The generalization of our proposed approach is that it is more conducive to achieving deployment on edge devices by utilizing small models with superior performance. Although the effectiveness of our proposed combined algorithm for Color-changing melon maturity detection has been validated, some things could be improved. The current algorithm is specific to Color-changing melon fruits, and the detection performance for more fruit varieties still needs to be proven. Meanwhile, this study only deals with the mature recognition part of Color-changing melon and does not mention the algorithms related to localization in the robotic picking behavior. In conclusion, our proposed algorithm can provide technical support for picking color-changing melons and some ideas for the automatic picking of melons, which need to be researched more in intelligent agriculture. In the future, we can explore other application scenarios, such as the detection of tomato fruits, to verify the applicability of the algorithm in other scenarios.

## Data availability statement

The raw data supporting the conclusions of this article will be made available by the authors, without undue reservation.

## Author contributions

GC: Formal analysis, Methodology, Supervision, Writing – original draft. YH: Conceptualization, Data curation, Methodology, Resources, Software, Writing – original draft, Writing – review & editing. HC: Validation, Visualization, Writing – review & editing. LC: Supervision, Validation, Writing – review & editing. JY: Investigation, Methodology, Writing – review & editing.
